# Non-alcoholic fatty liver disease and the gut microbiota in adolescents: is there a relationship?

**DOI:** 10.1186/s12887-024-05268-y

**Published:** 2024-11-29

**Authors:** Doaa El Amrousy, Heba El Ashry, Sara Maher, Yousef Elsayed, Samir Hasan

**Affiliations:** 1 Pediatric Department, Faculty of Medicine, Tanta University, Tanta, Egypt; 2 Tropical Medicine Departments, Faculty of Medicine, Tanta University, Tanta, Egypt; 3 Theodor Bilharz Research Institute, Cairo, Egypt; 4 Kasr El Ainy Medical School, Cairo University, Cairo, Egypt; 5 Immunology Lab, Theodor Bilharz Research Institute, Kornish El Nil Street, Giza, Egypt

**Keywords:** Adolescents, Metabolic dysfunction–associated steatotic liver disease, Gut microbiota

## Abstract

**Background:**

Despite the increasing prevalence of nonalcoholic fatty liver disease (NAFLD), the pathophysiology is still not fully understood. Recent evidence suggests that the gut microbiota may play a role in the pathophysiology of NAFLD and may also offer new therapeutic options.

**Methods:**

This prospective cross-sectional study included 100 consecutive newly diagnosed obese patients (BMI ≥ 95th percentile), aged 14–18 years with NAFLD (confirmed by ultrasound), persistently elevated levels of alanine aminotransferase (ALT) greater than 60 U/L for 1–6 months, and 100 healthy controls. We evaluated changes in the gut microbiota in NAFLD adolescents compared with healthy controls.

**Results:**

According to the multiple logistic regressions, the variables associated with NAFLD were the presence of Clostridium difficile, the presence of Salmonella spp., a greater abundance of Bifidobacterium and Prevotella, and a lower abundance of Lactobacillus.

**Conclusion:**

Changes in the gut microbiota occur in adolescents with NAFLD compared with healthy individuals, which may be useful for identifying youths who are amenable to gut microbiota-based interventions.

**Clinical trial number:**

Not applicable.

## Introduction

Nonalcoholic fatty liver disease (NAFLD), is the most common cause of hepatitis in children and adults [1]. NAFLD encompasses a spectrum of diseases, from simple hepatic steatosis to nonalcoholic steatohepatitis (NASH). NAFLD is a chronic liver disease that is more likely to progress to cirrhosis when NASH is present. NAFLD is an increasingly common indication for liver transplantation among adult patients and is the leading indication for liver transplantation among adult women [2]. NAFLD is estimated to affect 25% of the global population [3]. The prevalence of NAFLD in the pediatric population is estimated to be 13% (9.8% adjusted), with an age-dependent increase in prevalence from less than 1% among children aged 2–4 years to 17% among adolescents [4]. Furthermore, the incidence of NAFLD in children has dramatically increased from 36/100,000 in 2009 to 58.2/100,000 in 2018, in parallel with the worsening pediatric obesity epidemic (5–6), with a low rate of adherence to NAFLD screening guidelines; however, the true prevalence and incidence of NAFLD among children are likely underappreciated (6–7).

The term NAFLD is used to describe the entire broad spectrum of liver diseases that range from hepatic steatosis to steatohepatitis, fibrosis, and cirrhosis. Disease progression is considered a multihit process. The “two-hit NAFLD” hypothesis was first suggested by Day and James [8], with the initial “hit” resulting in the development of hepatic steatosis, whereas the second “hit” involves oxidative stress and lipid peroxidation, ultimately causing steatohepatitis. Multiple mechanisms have been proposed for each of these “hits”, suggesting that NAFLD may be a “final common outcome” from an accumulated series of diverse insults to the liver. Recently, the intestinal microbiome has been implicated in contributing to each “hit”, which is capable of both triggering and exacerbating NAFLD pathophysiology.

The intestinal tract is home to a vast microbial ecosystem that lives in a symbiotic relationship with its host (9–10). The intestinal microbiota clearly has a profound impact on human health and disease, but the mechanisms by which this occurs are not completely understood (11–12).

There are multiple interdependent interactions between the intestinal microbiome and the liver. Enterohepatic circulation involves communication between the liver and gut, whereby the liver secretes bile/bile acids and other metabolites through the common bile duct into the small intestine. The majority of primary bile acids are reabsorbed in the small intestine and returned to the liver through the portal vein. Commensal bacteria further metabolize the remaining primary bile acids into secondary bile acids, which, along with other microbial products (e.g., microbe-associated molecular patterns (MAMPs) such as flagellin and LPS) and dietary metabolites, can be reabsorbed by the epithelium and returned to the liver by the hepatic portal system, provided that they are not excreted. Intact bacteria also gain access to the liver through the portal vein. The liver is considered a microbial “firewall” for pathogens and commensals, clearing bacteria from circulation and preventing systemic spread and infection (13–14). The majority of bacteria are cleared from the liver after being engulfed by Kupffer cells, which are resident liver macrophages. Disruption of this bidirectional communication, through either damage or disease of the intestinal tract or liver, will likely result in reciprocal organ dysfunction. Liver dysfunction will alter the composition of bile and metabolic components delivered to the small intestine, thus changing the intestinal microenvironment and resulting in a perturbed microbial composition [15]. Reciprocally, intestinal dysbiosis also generates altered microbial metabolites (SCFAs, acetaldehyde, and secondary bile acids) and bacterial products (LPS, LTA, CpG DNA and other MAMPs) that are delivered to the liver via the portal vein, resulting in altered hepatic function [15]. Furthermore, intestinal dysbiosis driven by diet, genetics, or xenobiotics can loosen tight junctions, allowing increased transport of bacteria and altered bacterial byproducts to the portal circulation. Thus, dysbiosis associated with liver disease may be both a cause and an effect of liver damage.

Few studies have evaluated changes in gut microbiota among adolescents with NAFLD, and existing research presents conflicting results, likely due to variations in genetic and dietary habits. To our knowledge, this is the first study to correlate a microbiome signature specifically for NAFLD in adolescent children in the Middle East. Our aim was to assess the differences in gut microbiota between adolescent NAFLD patients and healthy subjects.

## Subjects and methods

This prospective cross-sectional study was carried out at the Pediatric and Gastroenterology departments of Tanta University Hospital between June 2022 and April 2023. One hundred consecutive newly diagnosed obese patients (BMI ≥ 95th percentile) 14–18 years old with NAFLD (ultrasound proven) and persistently elevated alanine aminotransferase (ALT) levels greater than 60 U/L for 1–6 months were included and constituted group I. One hundred matched volunteers who attended our outpatient clinics for upper respiratory tract infections were included in the study after complete resolution and constituted the control group (Group II).

### Inclusion criteria

Obese adolescents aged 14–18 years with ultrasound-proven NAFLD and high ALT.

### The exclusion criteria were as follows

previous/current history of other gastrointestinal tract GIT diseases, such as irritable bowel disease; inflammatory bowel disease; celiac disease; chronic diseases, such as cardiac, metabolic (including type 1 and 2 diabetes); renal, or hepatic diseases; recent diarrhea; and recent use of drugs (antibiotics, antiparasitics, pre/probiotics, vitamins, antacids, laxatives) within the last 3 months.

All included subjects underwent thorough clinical examination, including anthropometric measurements such as height, weight, waist circumference (WC), hip circumference (HC), waist‒hip ratio (W/H), and body mass index (BMI). BMI was calculated as weight (kg)/(height in meters)^2^. Subjects with a BMI ≥ 95th percentile were classified into the obese group, and subjects with a 5th percentile < BMI < 85th percentile were classified into the normal-weight group. Blood pressure was measured at the left arm three times consecutively with 1-min intervals after at least 5 min of rest in the seated position; the three readings were averaged for analysis. The subjects completed questionnaires on lifestyle, bowel habits, and dietary intake and submitted them at the hospital visit.

All laboratory data, including hepatitis B and C marker data, were drawn. All the subjects were assessed after overnight fasting for at least 10 h for total cholesterol (TC), triglyceride (TG), low-density lipoprotein (LDL), and high-density lipoprotein (HDL) levels. They were measured on an automatic analyzer (Hitachi 7080; Tokyo, Japan). Fasting glucose, 2-h postprandial glucose, and fasting serum insulin levels were also measured. The hemoglobin A1c (HbA1c) level was determined via a D-100 system (Bio-Rad Laboratories, Hercules, CA, USA). Insulin resistance and beta-cell function were evaluated via homeostasis model assessment methods (HOMA-IR and HOMA-%B, respectively). HOMA-IR was calculated as (insulin (µIU/mL) × glucose (mg/dL)/405), and HOMA-%B was calculated as (20 × insulin (µIU/mL)/(glucose (mg/dL)/18-3.5) [16]. Alanine aminotransferase (ALT), aspartate aminotransferase (AST), gamma-glutamyl transferase (GGT), complete blood count, and high-sensitivity C-reactive protein (hs-CRP) were measured according to standard protocols.

### Assessment of socioeconomic and housing conditions

Data regarding family composition and housing conditions were obtained through direct interviews with parents/caregivers.

#### Dietary assessments

The children and their parents received specific training from a dietitian to describe correctly all the foods and the quantities consumed, including the name/brand of the consumed food, recipes of dishes, methods of preparation or cooking, and portion sizes. After training with the dietitian, the participants filled out everything the subjects ate and drank for 3 days: 2 weekdays and 1 weekend day. Using data from the dietary records, nutrient intakes were calculated by a dietitian via the Computer-Aided Analysis Program.

### Neonatal data

Data on childbirth, gestational age at birth, weight and length at birth, and total duration of breastfeeding were collected.

### Liver ultrasonography

Liver ultrasonography was carried out by an expert radiologist who was not a part of the study and used an Acuson S2000 system (Siemens) with linear and convex transducers of 4–14 MHz to diagnose the presence of NAFLD and to define its stage. Grade 0 was defined as normal liver echo-texture with no steatosis; Grade I was defined as slight and diffuse increases in fine parenchymal echoes with normal visualization of the diaphragm and portal vein borders, which means mild steatosis; Grade II was defined as moderate and diffuse increases in fine echoes with slightly impaired visualization of the diaphragm and portal vein borders, which means moderate steatosis; and Grade III was defined as fine echoes with poor or no visualization of the diaphragm, portal vein borders, and posterior portion of the right lobe, which means severe steatosis [17]. The intraobserver variability was good, with an intraclass correlation coefficient (ICC) of 92% (95% CI: 88–95%).

### Stool collection and storage

Stool samples were collected once from all controls and patients before any treatment for *H. pylori* was started. Fecal samples were collected at home with the help of a stool sampling kit. The stool sampling kit consisted of a plastic lining to cover the toilet, two stool sample tubes with spoons, two plastic bags, and a clipping system for safe closure of the outer bag. The collected samples were stored at home between 4 °C and 8 °C and transferred to the laboratory within 24 h. Approximately one gram of feces from the collected samples was transferred to a sterile microcentrifuge tube containing ASL buffer from the QIAamp DNA Stool Mini Kit (Qiagen, Hilden, Germany) and then frozen at -20 °C until DNA extraction.

### Assessment of bacteria via real-time PCR

For real-time PCR, primers were designed on the basis of 16 S rDNA gene sequences of a variety of intestinal microbiota obtained from GenBank (http://www.ncbi.nlm.nih.gov/genbank/). The sequences were aligned via the Lasergene Software Package (DNASTAR, Madison, WI, USA), allowing regions conserved in all species to be chosen for annealing. All the designed primers were tested for their specificity via the Basic Local Alignment Search Tool (BLAST) software (http://www.ncbi.nlm.nih.gov/blast/), which has been proven to be specific for their purposes. The primers used were designed to identify the following constituents of the gut microbiota [18]: total Eubacteria, Bacteroidetes and Firmicutes phyla; Bifidobacterium spp.; *Bacteroides fragilis*; *Clostridium difficile*; *Clostridium perfringens*; *Enterococcus faecalis*; Lactobacillus spp.; *Staphylococcus aureus*; *Escherichia coli*; Salmonella spp.; and Methanobrevibacter smithii. The DNA in all the fecal samples was subjected to a real-time PCR trial to investigate the constituents of the gut microbiota of each participant. All reactions were performed in duplicate, each with a final volume of 10 L containing 5 L of Rotor-gene SYBR Green PCR Master Mix (Qiagen, Hilden, Germany), 0.2 L (10 pmol/L) of forward and reverse primers for each constituent of the gut microbiota, 0.5 L of the DNA sample (20 ng/L) and 4.1 L of DEPC water (Qiagen, Hilden, Germany). Thermocycling was performed on a Rotor-gene Q PCR cycler (Qiagen, Hilden, Germany) under the following conditions: 5 min at 95 °C, followed by 40 cycles of 95 °C for 10 s and 60 °C for 15 s. The cycle of dissociation for the melting curve was 95 °C for 1 min, and a step for producing the melting curve progressed from 70 °C to 95 °C with gradual temperature increases of 1 °C/s. The standard curve for all analyses was produced by amplifying a TOPO-TA plasmid (Invitrogen) carrying the gene fragment for each bacterium previously amplified via conventional PCR; its specificity was confirmed by sequencing and alignment via BLAST software. Knowing the molecular mass of both the plasmid and the inserted gene, the number of copies of the inserted gene was calculated according to the following formula: mass in Daltons (g/mol) = (plasmid size plus size of the gene inserted in base pairs [bp]). (330 Da × 2 nucleotides/bp). Knowing the number of copies/g and the concentration of plasmid DNA, it is possible to calculate the number of copies present in the real-time PCR and establish values for the standard curve. The results were expressed in colony forming units/g feces (CFU/g), and the number of 16 S rRNA genes (or copies of the quantified gene when different from the 16 S rRNA gene) was used as the CFU count [19]. A reaction containing all reagents except for the DNA sample was used as a negative control.

### Detection of serum cytokines by enzyme-linked immunosorbent assay (ELISA)

Serum samples were collected from all patients, and the levels of cytokines (TNF-α, Il-17, Il-10, Il-12, and Il-23) were measured via ELISA kits. The absorbance of the samples was measured at 450 nm via an ELISA reader (MultiscanTMFC, Thermo Fisher, USA), and the concentration of each cytokine was calculated on the basis of the standard curve.

### Statistical analysis

Power analysis was performed via the G*Power program, which revealed that 97 patients in each group were required to achieve a power of 90%. All the quantitative data are expressed as the means ± standard deviations and medians (min‒max). Categorical variables are expressed as percentages. The data were tested for a normal distribution via the Kolmogorov‒Smirnov test. Differences between the means of more than two groups were analyzed by one-way analysis of variance (ANOVA) followed by post hoc analysis. Student’s t test was used in the case of a normal distribution of values, and the Mann‒Whitney U test was used in the case of a nonnormal distribution. Spearman correlation analysis was used to evaluate the relationships between electromechanical and clinical parameters. All the analyses were conducted via SPSS V.20 (SPSS, Chicago. IL, USA). A statistically significant difference was considered if *p* < 0.05.

## Results

This study included one hundred consecutive newly diagnosed obese patients (BMI ≥ 95th percentile) 14–18 years old with NAFLD (ultrasound proven) and persistently elevated alanine aminotransferase (ALT) levels greater than 60 U/L for 1–6 months; these patients composed the patient group (Group I). One hundred matched volunteers who attended our outpatient clinics for upper respiratory tract infections were included in the study after complete resolution and constituted the control group (Group II).

The demographic and lifestyle data of the included groups are presented in Table 1, which shows nonsignificant differences between the patient group and the control group in terms of age (*p* = 0.72) and sex (*p* = 0.81), whereas the patient group presented significantly greater weight (p 0.003), weight z score (p **<** 0.001), height (*p* = 0.001), BMI (p = < 0.001), BMI z score (p = < 0.001), systolic blood pressure (*p* = 0.04), diastolic blood pressure (*p* = 0.01), waist circumference (p = < 0.001), midarm circumference (**<** 0.001), and thigh circumference (p = < 0.001) than did the control group. There was no significant difference between the 2 groups regarding mode of delivery (*p* = 0.5). Compared with healthy controls, patients with NAFLD spent significantly less time exercising during the day (*p* = 0.01) and spent significantly more time watching TV/using electronic devices (*p* = 0.04).

**Table 1:** Characteristics of the study participants

	Group I (*n* = 100)	Group II (*n* = 100)	*P* value
	NAFLD patients	Healthy control	
Age (years)	16.2±2.6	15.8 ± 1.9	0.72
Sex (male, n %)	54 (54%)	52 (52%)	0.81
Anthropometric measurements			
Weight (kg)	60.5±8.2	37.4 ± 10.5	**0.003**
Weight (z score)	2.6±0.6	0.8±0.6	**< 0.001**
Height(cm)	144.4 ± 8.3	139.5±9.2	**0.001**
Height(z score)	0.9 ± 0.6	0.8±0.7	0.1
BMI(kg/m^2^)	26.2 (24.3;28.1	17.1 (15.8–18.0)	**< 0.001**
BMI(z score)	2.9 (2.3;3.1)	0.7 (0.5;0.9)	**< 0.001**
Systolic blood pressure(mmHg)	112 (98;121)	96.6 (92.2;110.2)	**0.04**
Diastolic blood pressure(mmHg)	72.1(60.5;72.0)	61.2(60.0;64.1)	**0.01**
Waist circumference(cm)	79.2 (73.5;84.1)	59.2 (54.8;62.1)	**< 0.001**
Waist/height ratio	0.7 ± 0.2	0.5 ± 0.1	**< 0.001**
Midarm circumferences	28.3 ± 3.1	22.4± 2.9	**< 0.001**
Hip circumferenxes	87.9 ± 9.1	72.5 ± 7.1	**< 0.001**
Thight circumference(cm)	50.6 (46.2;55.1)	40.7 (36.2;43.5)	**< 0.001**
Delivery type			
Vaginal: cesarean	47:53	49:51	0.5
Lifestyle pattern			
Study time after school			
≤ 1 h	55 (55%)	53 (53%)	0.92
>1 h	45 (45%)	47 (47%)	
Exercise time during the day			
≤ 1 h	72 (72%)	58 (58%)	**0.01**
>1 h	28 (28%)	42 (42%)	
Time watching TV/using electronic			
Devices			**0.04**
≤ 2 h	36 (36%)	42 (42%)	
> hour	64 (64%)	58 (58%)	

∗Data are expressed as the mean ± standard deviation, median (interquartile range), or number (%). † Significant p values (< 0.05) are shown in bold

The daily energy and nutritional intakes of both groups are recorded in Table 2, which shows significantly greater parameters in NAFLD patients than in healthy controls regarding total energy intake (p = < 0.001), protein (p = < 0.001), fat (p = < 0.001) and carbohydrate intake (p = < 0.001). While there were no significant differences in the mean total daily intake of calcium (*p* = 0.77), phosphorus (*p* = 0.81), iron (*p* = 0.3), folic acid (*p* = 0.55), B-carotene (*p* = 0.71), retinol (*p* = 0.44), riboflavin (*p* = 0.61), vitamin c (*p* = 0.73), vitamin d (*p* = 0.98), fiber (0.26) and saturated fatty acids (*p* = 0.1) between the two groups, the patient group had significantly greater mean daily intake of sodium (p = < 0.001), potassium (*p* = 0.01), zinc (*p* = 0.04), thiamine (*p* = 0.03), niacin (p = < 0.001), vitamin B6 (p = < 0.001), vitamin E (*p* = 0.002), cholesterol (p = < 0.001), total fatty acids (*p* = 0.001) and polyunsaturated fatty acids (*p* = 0.01) than did the healthy control group.

**Table 2:** Daily energy and nutritional intake of the participants

	Group I(*n* = 100) NAFLD patients	Group II(*n* = 100) Healthy control	*P* value
Energy(kCal)	2473 ± 602	1688± 408	**< 0.001**
Protein(g)	79.3 ± 28.1	60.5 19.2	**< 0.001**
Fat(g)	62.1 ± 27.8	43.3 ± 15.9	**< 0.001**
Carbohydrate(g)	301.6 ± 2.5	255.8 ± 59.8	**< 0.001**
Calcium(mg)	510.2± 199.7	496.5± 311.7	0.77
Phosphorus(mg)	901.4 ± 388	875.9 ± 366.1	0.81
Iron(mg)	13.6 ± 4.5	11.9 ± 4.7	0.3
Sodium(mg)	4077.4 ± 973.5	2911.3 ± 1005.7	**< 0.001**
Potasium(mg)	2375.7 811.3	2135.5 801.5	**0.01**
Zinc(mg)	10.2 ± 3.1	9.5 ± 2.0	**0.04**
Folic acid(µg)	450.3± 177.9	431.6 ± 208.5	0.55
B-Carotene (µg)	3121.6 1022.6	3088.4 ± 1134.7	0.71
Retinol (µg)	153.7 ± 94.1	122.7 ± 85.3	0.44
Thiamine(mg)	1.3± 0.7	1.2 ± 0.4	**0.03**
Riboflavin(mg)	1.5 ± 0.6	1.4 ± 0.4	0.61
Niacin(mg)	19.0 ± 7.2	13.5 ± 4.8	**< 0.001**
Vitamin B6(mg)	1.8 ± 0.4	1.1 ± 0.3	**< 0.001**
Vitamin C(mg)	91.3 14.7	93.6 16.2	0.73
Vitamin D (µg)	3.5 ± 0.9	2.9 1.1	0.98
Vitamin E(mg)	17.9 ± 9.2	12.6 ± 6.2	**0.002**
Fiber(g)	17.3 2.5	16.1 ± 3.0	0.26
Cholesterol(mg)	446.2 ± 198.7	261.2 152.8	**< 0.001**
Total Fatty Acids(mg)	40.2 ± 23.6	26.3 ± 7.9	**< 0.001**
Saturated Fatty Acids(mg)	13.5 ± 5.8	10.1 ± 2.7	0.1
Polyunsaturated Fatty Acids(mg)	12.1 ± 4.9	6.3 ± 3.2	**0.01**

The data are expressed as the means ± standard deviations

Significant p values (< 0.05) are shown in bold

The laboratory parameters of both groups are shown in Table 3. Compared with the control group, the patient group had significantly greater mean ALT (p = < 0.001), total cholesterol (*p* = 0.01), triglyceride (p = < 0.001), LDL-cholesterol (p = < 0.001), and uric acid (*p* = 0.04) levels and significantly lower HDL-cholesterol (*p* = 0.01) levels. Compared with the control group, the patient group presented significantly greater mean levels of fasting blood glucose (*p* = 0.03), serum insulin (*p* ≤ 0.001) and HOMA-IR (*p* ≤ 0.001), with nonsignificant differences in HbA1c (*p* = 0.35) between the two groups. While the patient group presented significantly lower levels of 25-OH vitamin D than did the control group (*p* = 0.03), there were no significant differences between the two groups in terms of iron status, including serum iron (*p* = 0.6), TIBC (*p* = 0.8), transferrin saturation (*p* = 0.74) and ferritin levels (*p* = 0.55). Compared with those in the healthy control group, the levels of all the inflammatory markers, including hs-CRP (*p* ≤ 0.001), uric acid (*p* = 0.04), TNF-α (*p* = 0.003), IL-17 (*p* = 0.01), IL-10 (*p* = 0.04), IL-12 (*p* = 0.01) and IL-23 (*p* ≤ 0.001), were significantly greater. Compared with the control group, the patient group presented significantly greater mean total WBC counts (*p* = 0.04), total neutrophil counts (*p* = 0.02), neutrophil percentages (*p* = 0.02) and neutrophil/lymphocyte ratios (*p* = 0.01), with nonsignificant differences in other CBC components, including hemoglobin levels (*p* = 0.4), platelet counts (*p* = 0.9), MPV (*p* = 0.77), PDW (*p* = 0.53), lymphocyte counts (*p* = 0.6), lymphocyte percentages (*p* = 0.1), monocyte counts (*p* = 0.8), eosinophil counts (*p* = 0.9) and basophil counts (0.82).

**Table 3:** Laboratory parameters of the participants and abdominal ultrasonic stages of steatosis in NAFLD patients

	Group I(*n* = 100) NAFLD patients	Group II(*n* = 100) Healthy control	*P* value
Fasting blood Glucose(mg/dL)	104.4 ± 8.3	96.5 ± 5.1	**0.03**
ALT(IU/L)	72.8 ± 11.6	12.7 ± 5.7	**< 0.001**
AST(IU/L)	24.3 ± 7.3	20.4 ± 9.2	0.4
Total cholesterol(mg/dl)	186.2 ± 30.7	155.7 ± 17.5	**0.01**
Triglycerides(mg/dL)	92.2 ± 14.7	52.5 ± 11.6	**< 0.001**
HDL-cholesterol(mg/dL)	56.3 ± 10.9	64.7 ± 11.2	**0.01**
LDL-cholesterol(mg/dL)	118.3 ± 19.4	91.1± 21.5	**< 0.001**
hs-CRP(mg/L)	5.1 ± 1.1	0.6 ± 0.3	**< 0.001**
Uric acid(mg/L)	4.9 ± 1.5	4.0 ± 0.9	**0.04**
Iron(µg/dL)	97.2 ± 29.5	102.6 ± 36.8	0.6
TIBC(µg/dL)	338.3 ± 40.5	327.8 ± 32.7	0.8
Transferrin saturation (%)	27.5± 7.3	30.9 ± 11.3	0.74
Ferritin(ng/mL	41.2 ± 18.4	35.2 ± 16.9	0.55
25-OH vitamin D(ng/mL	11.2 ± 4.0	16.3 ± 7.2	**0.03**
Insulin(µU/mL)	12.8 ± 3.7	4.7 ± 2.5	**< 0.001**
HOMA-IR	3.6 ± 1.1	1.3 ± 0.8	**< 0.001**
Hemoglobin(g/dL)	13.8 ± 1.3	14.1 ± 1.1	0.4
HbA1c (%)	5.4 ± 0.8	5.2 ± 0.5	0.35
Platelet count(×10^3^/µL)	255.8 ± 48.3	245.7 ± 50.3	0.9
MPV(fL)	7.2 ± 0.6	7.1 ± 0.8	0.77
PDW (%)	46.6 ± 2.8	44.9 ± 1.3	0.53
WBC count(×10^3^/µL)	7.4 ± 1.4	5.9 ± 1.1	**0.04**
Neutrophil count	3.8 ± 2.1	2.6 ± 1.7	**0.02**
Neutrophil %	51.2 ± 7.5	42.8 ± 2.6	**0.02**
Lymphocyte count(×10^3^/µL)	2.9 ± 0.8	2.6 ± 1.2	0.6
Lymphocyte %	37.8 6.2	44.2 7.0	0.1
Monocyte count(×10^3^/µL)	0.4 ± 0.1	0.3 ± 0.1	0.8
Eosinophil count(×10^3^/µL)	0.2 ± 0.1	0.2 ± 0.1	0.9
Basophil count(×10^3^/µL)	0.1	0.1	0.82
Neutrophil/lymphocyte ratio	1.3 ± 0.4	0.9 ± 0.2	**0.01**
TNF-α(pg/ml)	22.6 ± 5.8	16.5 ± 3.6	**0.003**
IL-17(pg/ml)	14.7 ± 5.8	8.65± 3.08	**0.01**
IL-10(pg/ml)	9.4 ± 3.6	7.8 ± 1.5	**0.04**
IL-12(pg/ml)	74.1 ± 26.8	55.4 ± 22.6	**0.01**
IL-23(pg/ml)	61.4 ± 19.7	43.7 ± 18.6	**< 0.001**

The data are expressed as the means ± standard deviations, and significant p values (< 0.05) are shown in bold

ALT: alanine aminotransferase; AST: aspartate aminotransferase; HDL: high-density lipoprotein; LDL: low-density lipoprotein; hs-CRP: high-sensitivity C-reactive protein; TIBC: total iron binding capacity; HbA1c: glycated hemoglobin; HOMA-IR: homeostasis model assessment of insulin resistance; MPV: mean platelet volume; PDW: platelet distribution width; WBC: white blood cell; TNF: tumor necrosis factor; IL: interleukin

The prevalence of gut microbiota in the studied groups is described in Table 4. *Clostridium difficile* and Salmonella spp. were significantly more common in the patient group (Group I) than in the control group (Group II) (*p* values of 0.01 and 0.02, respectively). The abundances of Cladosporium perfringens (slightly greater in group II), *Enterococcus faecalis* (lower in group II), and *Staphylococcus aureus* (slightly lower in group I) were not significantly different between the two groups. The phyla Bacteroides and Firmicutes, the genera Bifidobacterium spp., Lactobacillus spp., and the species *Bacteroides fragilis*, *Escherichia coli* and Prevotella copri were present in all the samples analyzed.

**Table 4:** Prevalence of the gut microbiota in the studied groups

*N* (%)	Group I (*n* = 100)	Group II (*n* = 100)	OR (95% CI)	*p*
	NAFLD patients	Healthy control		
Total Eubacteria	100	100	NC	NC
Actinobacteria	-	-	-	-
Bifidobacterium spp.	100	100	NC	NC
Bacteroidetes	100	100	NC	NC
*Bacteroides fragilis*	100	100		
Firmicutes	100	100	NC	NC
*Clostridium difficile*	61	44	3.3 (1.06–8.4)	**0.01**
*Clostridium perfringens*	38	64	1.8 (0.51–8.5)	0.42
*Enterococcus faecalis*	16	13	0.41 (0.08–1.33)	0.22
Lactobacillus spp.	100	100	NC	NC
*Staphylococcus aureus*	46	52	0.87 (0.41–2.08)	0.68
Proteobacteria	-	-	-	-
*Escherichia coli*	100	100	NC	NC
Salmonella spp.	58	30	2.08 (0.82–5.9)	**0.02**
Euyarchaeota	-	-	-	-
Prevotella copri	100	100	NC	NC

Chi-square tests

NC = not calculatedP significant < 0.05

With respect to the counts of Eubacteria and the phyla, genera and species analyzed, adolescents with NAFLD (group I) presented significantly lower total Eubacteria counts (*p* < 0.001), significantly lower *Bacteroides fragilis* counts (*p* < 0.001), significantly lower Lactobacillus spp. counts (*p* < 0.001), significantly lower *Escherichia coli* counts (*p* = 0.02) and significantly greater Prevotella counts (*p* = 0.03). No statistically significant differences in *Clostridium difficile* (*p* = 0.76), *Clostridium* perfringens (*p* = 0.57), *Enterococcus faecalis* (*p* = 0.23) or *Staphylococcus aureus* (*p* = 0.2) were detected between the NAFLD patient group and the control group. The median counts of Bifidobacterium spp. (*p* = 0.09) and Salmonella spp. (*p* = 0.1) were greater in the NAFLD patient group than in the control group, but the difference was not statistically significant (Table 5).

**Table 5:** Counts (colony forming units/gram of feces, CFU/g) of the gut microbiota in the studied groups

	10^*n*^ CFU/g	Group I (*n* = 100) NAFLD patients	Group II (*n* = 100) Healthy control	*p*
Eubacteria	× 10^13^	0.8 (0.00-0.16)	5.88 (0.6–28.4)	**< 0.001**
Bacteroidetes	× 10^9^	0.59 (0.17–2.38)	1.43 (0.43–2.92)	0.44
Firmicutes	× 10^8^	0.38 (0.06–2.37)	0.84 (0.29–2.66)	0.35
*Bacteroides fragilis*	× 10^9^	0.3 (0.00-0.58)	3.6 (0.07–45.1)	**< 0.001**
Bifidobacterium spp.	× 10^6^	1.32 (0.49–4.14)	0.35 (0.07–2.31)	0.09
*Clostridium difficile*	× 10^3^	0.83 (0.24–3.04)	0.66 (0.00-4.38)	0.76
*Clostridium perfringens*	× 10^5^	1.42 (0.16–4.35)	1.29 (0.04–8.82)	0.57
*Enterococcus faecalis*	× 10	0.00	0.00	0.23
Lactobacillus spp.	× 10^8^	0.04 (0.00-0.09)	0.39 (0.2–1.42)	**< 0.001**
*Staphylococcus aureus*	× 10^4^	0.06 (0.00-1.55)	0.67 (0.00-11.4)	0.2
*Escherichia coli*	× 10^9^	0.15 (0.04–1.20)	1.39 (0.24–8.44)	**0.02**
Salmonella spp.	× 10^3^	0.13 (0.00-1.35)	0.00 (0.00-0.21)	0.1
Prevotella copri	× 10^8^	0.41 (0.00-0.17)	0.04 (0.04–1.10)	**0.03**

Wilcoxon‒Mann‒Whitney test: median (25th and 75th percentiles)

P significant < 0.05

Multiple logistic regression was conducted (Table 6) with the occurrence of NAFLD as the outcome variable. The dependent variables included in the model were as follows: (1) presence or absence of *Clostridium difficile* and Salmonella spp.; (2) higher abundance (above the 50th percentile in the population studied) of *Bacteroides fragilis*, Bifidobacterium spp., *Escherichia coli*, and Prevotella; and lower abundance of Lactobacillus spp. According to the multiple logistic regression, the variables associated with NAFLD were the presence of *Clostridium difficile*, presence of Salmonella spp., greater abundance of Bifidobacterium and Prevotella, and lower abundance of Lactobacillus.

**Table 6:** Multiple logistic regression analysis with NAFLD patients

	OR	95% CI	*p*
*Bacteroides fragilis* Low: ≤4.22 × 10^8^ versus high: >4.22 × 10^8^	0.79	0.20–3.88	063
Bifidobacterium spp Low: ≤6.75 × 10^5^ versus high: >6.75 × 10^5^	7.44	3.19–35.28	**0.03**
*Clostridium difficile* Presence versus absence	4.78	1.4-22.51	**0.01**
Lactobacillus spp. Low: ≤4.22 × 10^8^ versus high: >4.22 × 10^8^	0.2	0.04–0.68	**0.02**
*Escherichia coli* Low: ≤4.22 × 10^8^ versus high: >4.22 × 10^8^	2.7	0.79–13.07	0.34
Salmonella spp. Presence versus abscence	1.57	0.11–2.43	**0.02**
Prevotella copri Low:≤4.22 × 10^8^ versus high > 4.22 × 10^8^	1.67	0.59–6.15	**0.01**

P significant < 0.05

## Discussion

Among the intestinal microbiomes studied in our study, we detected greater abundances (above the 50th percentile in the population studied) of *Bacteroides fragilis*, Bifidobacterium spp., *Escherichia coli*, Lactobacillus spp. and Prevotella spp. in adolescents with NAFLD than in healthy controls. According to the multiple logistic regressions, the variables associated with NAFLD were the presence of *Clostridium difficile*, the presence of Salmonella spp., a greater abundance of Bifidobacterium and Prevotella, and a lower abundance of Lactobacillus. The studied species was selected according to our previous work described its abundance in Egyptian population [20].

There is growing interest in investigating the relationships between the intestinal microbiome/dysbiosis and different human diseases, especially gastrointestinal diseases. Multiple pediatric disorders, including inflammatory bowel disease (IBD) and T1DM, are associated with gut dysbiosis and increased gut permeability. Multiple human and animal studies have demonstrated a significant role for the intestinal microbiome in the disease pathophysiology of chronic inflammatory diseases, including IBD, T1DM and NAFLD [21–24]. This relationship has been widely investigated in adults with NAFLD, but few studies on pediatric NAFLD are available, with conflicting results. To the best of our knowledge, this is the first work evaluating the relationship between changes in the gut microbiota and NAFLD in adolescent patients in the Middle East, which are considered special ethnic groups with different food habits.

There are multiple animal and human studies with different hypotheses explaining the mechanisms that involve the gut microbiota in the pathogenesis of NAFLD. Hepatic steatosis is the initial step in the development of NAFLD. The intestinal microbiome can lead to hepatic steatosis in different ways, and the intestinal microbiota can metabolize dietary choline with subsequent choline deficiency, leading to the progressive development of hepatic steatosis, as choline is essential for hepatic lipid metabolism [25]. The intestinal microbiota can induce de novo hepatic lipogenesis by promoting the absorption of dietary monosaccharides [26], acetate production through fructose fermentation [27] and endogenous alcohol production [28].

In 2013, Zhu et al. evaluated 3 groups of children: a NASH group (22 patients), an obese group with normal ALT levels and a normal control group. Those authors reported that patients with NASH had increased serum ethanol levels, which was attributed to the increased abundance of Escherichia species with the ability to produce alcohol during anaerobic fermentation [29], in parallel with our findings.

In 2015, Michail et al. compared 3 groups of subjects: 13 children with NAFLD with obesity associated with high ALT and ultrasound-proven hepatic steatosis, 11 obese children with normal ALT and hepatic ultrasound, and 26 nonobese controls. They reported higher ethanol levels in the stool and more abundant Prevotella bacteria in patients with NAFLD (similar to our findings) than other groups did [30].

In contrast to Michail et al., in 2017, Del Chierico et al. evaluated 53 children with NAFLD (nearly half with and half without) nonalcoholic steatohepatitis (NASH),8 obese children without NAFLD and 54 healthy nonobese controls and reported that Prevotella abundance was not significantly different between the 4 groups studied, with nonsignificant mean values of ethanol in stool between patients with NASH and controls. However, ethanol was elevated in 17% of patients with NAFLD and 25% of controls [31]. The differences in Prevotella abundance with our funding may be explained by differences in age (7–16 years in Del Chierico et al. compared with 14–18 years in our study) and differences in ethnic groups with different feeding habits.

In 2019, Schwartzmer et al. [32] evaluated children with NAFLD versus obese children who did not have NAFLD on the basis of clinical and liver histology evaluations. These authors reported that among children with NAFLD at the genus level, Lactobacillus and Oribacterium abundances were greater in patients with NASH, whereas Oscillibacter, Lactobacillus, Akkermansia, and Enterococcus abundances were greater in patients with NAFLD but not NASH.

Pediatric studies to date have consistently shown that NAFLD is associated with dysbiosis and the loss of alpha diversity in the intestinal microbiome. However, the differences in study design make it difficult to compare results and draw firm conclusions related to bacterial composition. In addition, geographic, race/ethnicity, genetic and dietary differences may contribute to the microbiome variations between study populations, further complicating study interpretation.

Although there are several controversies between different studies, Prevotella and Eschierichia spp. were found to be commonly associated with NAFLD severity in both pediatric and adult studies [33].

Recent work has shown that Prevotella is a highly gnomically diverse species whose abundance in the gut is driven by its ability to utilize distinct polysaccharides and is overall underrepresented in the microbiomes of Westernized populations. Functional distinctions between members of a single species are lost when analysis is limited to 16 S rDNA sequencing [34–36]. A greater focus on microbial genetic and metabolic potential, through examination of the microbial metagenome and metatranscriptome, could yield more consistent results with greater relevance to disease pathophysiology and better direct investigation of disease mechanisms.

This study has several important limitations. Since it is cross-sectional, we cannot draw any conclusions about the direction of associations between the gut microbiota and liver fat. The highlighted taxa may contribute to fatty liver or may be the result of obesity and fatty liver. We identified numerous taxa that may prove to be important in the pathophysiology of NAFLD, but larger longitudinal studies are necessary to further understand and confirm our findings In a similar study conducted by Schwimmer et al., 2019 in San Diego, USA, they found that a high abundance of Prevotella copri was associated with more severe fibrosis [32]. Another important limitation is that we evaluated one ethnic group, Egyptian adolescents, while others may have different microbiome taxa depending on genetic and feeding habits; however, our selection results in more harmonious evaluated groups and easier differentiation. Thousands of microbiomes inhabit the human intestine, and only a few numbers were evaluated in our study; however, on the basis of previous studies, the microbiome selected in our study is the most important bacteria suspected to play a role in the pathophysiology of NAFLD patients.

There are many strengths of this study. The study included a relatively large number of subjects (100 patients and 100 healthy controls), which makes the results highly applicable. Dietary information from a questionnaire designed specifically for children. Owing to the young age of the participants in this study, the confounding effect of alcohol intake and prescription medications is likely much lower than that in older populations.

Our results suggest that, in the future, the gut microbiota may offer the potential to help identify youth at risk for NAFLD or to identify youth who may be particularly amenable to microbiota-based interventions for NAFLD.

## Data Availability

No datasets were generated or analysed during the current study.

## Declarations

### Access to study data

All authors had access to the study data and reviewed and approved the final manuscript.
